# Tamoxifen effects on subjective and psychosexual well-being, in a randomised breast cancer study comparing high-dose and standard-dose chemotherapy

**DOI:** 10.1038/sj.bjc.6600294

**Published:** 2002-05-03

**Authors:** M J Mourits, I Böckermann, E G de Vries, A G van der Zee, K A ten Hoor, W T van der Graaf, W J Sluiter, P H Willemse

**Affiliations:** Department of Gynaecology, University Hospital Groningen, Hanzeplein 1, 9713 GZ Groningen, The Netherlands; Department of Medical Oncology, University Hospital Groningen, Hanzeplein 1, 9713 GZ Groningen, The Netherlands; Department of Endocrinology, University Hospital Groningen, Hanzeplein 1, 9713 GZ Groningen, The Netherlands

**Keywords:** tamoxifen, sexual function, side effects, chemotherapy

## Abstract

To evaluate the impact of tamoxifen on subjective and psychosexual well-being in breast cancer patients in relation to type of prior chemotherapy and menopausal status. Longitudinal interview study in breast cancer patients during and after adjuvant tamoxifen use. Menopausal status was defined by last menstrual period and serum oestradiol and FSH levels. Gynaecology outpatient clinic, Tertiary Referral Hospital, January 1995 to September 1999. Breast cancer patients <56 years of age, participating in a randomised trial comparing adjuvant high-dose (*n*=45) and standard-dose (*n*=53) chemotherapy, followed by radiotherapy and tamoxifen. Relative incidence and correlation of subjective and psychosexual symptoms during and after tamoxifen. During tamoxifen the most frequent complaints were hot flushes (85%), disturbed sleep (55%), vaginal dryness and/or dyspareunia (47%), decreased sexual desire (44%) and musculo-skeletal symptoms (43%). Disturbed sleep correlated with hot flushes (*P*<0.0005) and concentration problems (*P*<0.05). Decreased sexual interest correlated with vaginal dryness (*P*<0.0005) and/or dyspareunia (*P*<0.0005). In the high-dose group more patients became postmenopausal (95% *vs* 33%) and more patients reported symptoms than in the standard-dose group (*P*<0.05). After discontinuation of tamoxifen, symptoms decreased significantly. However, hot flushes, disturbed sleep and vaginal dryness persisted more often in patients who remained postmenopausal after high-dose chemotherapy (*P*<0.05). Overall, during tamoxifen patients reported many symptoms. More patients become postmenopausal after high-dose chemotherapy, and they remain often symptomatic after tamoxifen.

*British Journal of Cancer* (2002) **86**, 1546–1550. DOI: 10.1038/sj/bjc/6600294 www.bjcancer.com

© 2002 Cancer Research UK

## 

Tamoxifen plays an important role as adjuvant therapy after surgery for primary breast cancer in premenopausal and postmenopausal women (Early Breast Cancer Trialists' Group, 1998). In addition, tamoxifen is prescribed for the prevention of breast cancer in high risk women in the United States since the Breast Cancer Prevention Trial (BCPT) of the National Surgical Adjuvant Breast and Bowel Project (NSABP) showed a breast cancer risk reduction in healthy women over the age of 35 years at high risk for breast cancer (Fisher *et al*, 1998), although two European studies could not confirm these results (Powles *et al*, 1998; Veronesi *et al*, 1998).

Being a drug with both agonistic and antagonistic properties, tamoxifen can have oestrogen as well as anti-oestrogen effects on different sites and tissues, depending on the ambient oestradiol concentration. Side effects of tamoxifen have been studied mainly in postmenopausal women (No authors listed, 1985; Fisher *et al*, 1989; Love, 1989, 1992; Love *et al*, 1991; Ragaz, 1997), while only a few studies included also women under 50 years of age. Although an important component of quality of life, few studies have paid attention to the impact of tamoxifen on sexual functioning (Ganz *et al*, 1995, 1998; Day *et al*, 1999). Moreover, in younger women, induction of menopause as a result of high-dose chemotherapy might have a negative impact on quality of life and sexual functioning. The present study was performed to assess the impact of tamoxifen on breast cancer patients under 56 years of age following adjuvant standard- or high-dose chemotherapy. We collected prospective information on subjective side effects and sexual functioning during and after tamoxifen use and related symptoms to type of chemotherapy and menopausal status as defined by last menstrual period and serum levels of follicle stimulating hormone (FSH) and oestradiol.

## PATIENTS AND METHODS

### Patients

Between January 1995 and September 1995 all breast cancer patients <56 years of age with four or more tumour positive axillary lymph nodes treated at the Department of Medical Oncology, University Hospital Groningen, The Netherlands, and participating in the Dutch randomised study comparing standard-dose and high-dose chemotherapy, were referred to the Gynaecology Outpatient Clinic. After surgery, patients had been randomly assigned to receive either five cycles of standard-dose 5-fluorouracil (500 mg m^−2^), epirubicin (90 mg m^−2^) and cyclophosphamide (500 mg m^−2^) (FEC) or four cycles of FEC plus one cycle of high-dose chemotherapy (cyclophosphamide 6 g m^−2^, thiotepa 480 mg m^−2^ and carboplatin 1.6 g m^−2^), followed by peripheral blood progenitor stem-cell rescue. In both treatment arms chemotherapy was followed by locoregional radiotherapy and 2 years of daily 40 mg tamoxifen (de Vries *et al*, 1996). The study was approved by the Medical Ethical Committee of the University Hospital Groningen. All patients gave informed consent.

### Methods

Patients were seen after completion of chemotherapy, at 6 monthly intervals during tamoxifen treatment and again 3–6 months after cessation of tamoxifen. Symptoms during and after tamoxifen were compared, to evaluate the effect of tamoxifen on the symptoms. Patients were excluded for analysis if tumour relapse occurred. At each visit a structured interview on the basis of two self-reported questionnaires was performed by the same gynaecologist (MM). The interviewer is an experienced clinician and sexuologist, trained in taking interviews. Interview items were derived from, at that time available, reports on side effects of tamoxifen (No authors listed, 1985; Fisher *et al*, 1989; Love, 1989, 1992; Love *et al*, 1991; Powles *et al*, 1994). The first part of the interview contained items of physical, mood-related and psychological symptoms associated with tamoxifen as well as symptoms associated with menopause (Appendix 1). Depressive symptoms were defined as reporting three or more of the following: feeling miserable and sad, lack of enjoyment, loss of interest, loss of vitality, loss of initiative, life not worth living. The second part of the interview contained the major components of sexual functioning: sexual interest (desire and ability to enjoy sex), activity (frequency, ability to perform sexual activity), and pleasure or discomfort (pain, vaginal dryness) (Appendix 2) (McCoy and Davidson, 1985; Wilmoth, 1993; Greendale *et al*, 1996; Thirlaway *et al*, 1996). With every question, patients were asked to compare the present situation with the situation before the cancer treatment. Only patients with a sexual partner were interviewed about sexual functioning.

As ovarian failure plays a major role in well-being and sexual function (McCoy and Davidson, 1985; Greendale *et al*, 1996), a menstrual history was recorded and at each visit serum levels of FSH (Amerlite FSH Assay, Johnson & Johnson Clinical Diagnostics Ltd., Amersham, UK) and oestradiol were determined by radio-immunoassay, as described previously (Jurjens *et al*, 1975). As tamoxifen can result in amenorrhoea without ovarian failure (Mourits *et al*, 1999), menopause was defined as no menstrual periods in the preceding 12 months and FSH levels >30 nmol l^−1^ and oestradiol levels <0.10 nmol l^−1^.

### Statistics

Data analysis was performed using software (SPSS 9.0; SPSS Inc: Chicago, USA). Variance of paired symptom score differences were analysed using the test of Pitman. Difference was corrected for continuity. Comparability in frequencies was analysed using the χ^2^ test. Only *P*-values <0.05 were considered significant.

## RESULTS

### Patient characteristics

A total of 116 patients were referred. Eighteen patients refused to take part in the interviews. Ninety-eight patients were enrolled for interviews and their characteristics are summarised in Table 1

**Table 1:**
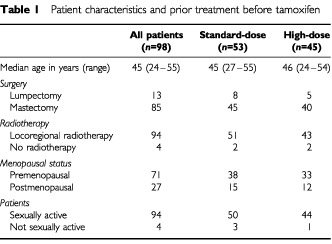
Patient characteristics and prior treatment before tamoxifen

. Forty-five patients had received high-dose chemotherapy and 53 standard-dose chemotherapy. Following surgery and chemotherapy, 94 patients received locoregional radiotherapy. None of the variables in Table 1 show an imbalance between patients in the two treatment arms. During the study four patients in the standard dose arm with severe depressive symptoms were offered a lower dose of daily 20 mg tamoxifen but were not excluded from the interview analysis. No other hormonal therapy was used during the study.

### Menopausal status

According to last menstrual period and serum hormone levels, 28% of all patients were postmenopausal at the start of tamoxifen, equally distributed over the treatment arms (Table 2

**Table 2:**
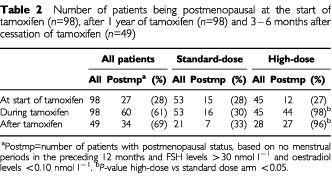
Number of patients being postmenopausal at the start of tamoxifen (*n*=98), after 1 year of tamoxifen (*n*=98) and 3–6 months after cessation of tamoxifen (*n*=49)

). One year after chemotherapy, during tamoxifen use, 61% of the patients were postmenopausal, more often in the high-dose group (98%) than in the standard-dose group (30%) (*P*<0.01). During the interview 3–6 months after tamoxifen was stopped, more patients were postmenopausal in the high-dose group (96%) than in the standard-dose group (33%) (*P*<0.01).

### Symptoms during tamoxifen

During the 2 years of tamoxifen treatment, the reported symptoms stayed stable except for the gastrointestinal complaints which disappeared after 1–3 months. We therefore show the symptoms at 1 year of tamoxifen use (Table 3

**Table 3:**
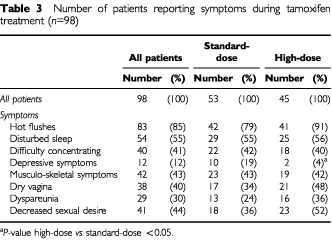
Number of patients reporting symptoms during tamoxifen treatment (*n*=98)

). Hot flushes were experienced by 86% of the patients during tamoxifen, not related to treatment arm (Table 3). Fifty-four patients (55%) suffered from disruption of sleep, i.e. awakening more than twice a night, which was not related to the type of chemotherapy received, but showed a strong relation with the occurrence of hot flushes (*P*<0.0005). Fifty-three out of 83 patients (64%) with hot flushes experienced disturbed sleep with night sweats, *vs* only one patient without hot flushes. Forty patients (41%) reported difficulty concentrating during tamoxifen treatment, a symptom which was not related to chemotherapy regimen. However, disruption of sleep was associated with concentration problems (*P*<0.05). Irritability was reported by 29% of the women and was related to difficulty concentrating (*P*<0.0005) and to disruption of sleep (*P*<0.05). Depressive symptoms were mentioned by 12% of the patients, 19% in the standard-dose and 4% in the high-dose arm (*P*<0.05) which led to discontinuation of tamoxifen use in two and dose reduction to 20 mg in four patients.

Gastrointestinal symptoms during tamoxifen use were mentioned by 43 patients (44%) at the beginning of tamoxifen, and subsided in all patients with 1–3 months after starting tamoxifen.

### Sexual functioning during tamoxifen

Ninety-four patients had a sexual partner during tamoxifen of whom 37 were pre- and 57 were postmenopausal after 1 year on tamoxifen. Results are shown in Table 3. Vaginal dryness during intercourse was mentioned by 40%, 34% in the standard-dose and 48% in the high-dose arm (ns). Decreased sexual desire was reported by 44% of the patients, which did not differ between patients in the standard-dose and high-dose arm, or between premenopausal and postmenopausal women. Decreased libido was strongly related to a dry vagina (*P*<0.0005) and dyspareunia (*P*<0.0005). These complaints led to decreased sexual activity in 28 out of 94 (30%) (10 pre- and 18 postmenopausal) patients. Five patients were unable to be sexually active due to painful intercourse or complete loss of sexual desire.

### Symptoms after tamoxifen

Eighty-two patients were seen 3–6 months following the end of tamoxifen treatment; 33 of them were not evaluable because of disease progression and other treatment of which 15 were in the high-dose and 18 in the standard-dose arm. Ten patients were still using tamoxifen and six had withdrawn from the study. As a result 49 patients were disease-free during the interview after completion of tamoxifen treatment, 21 in the standard-dose arm and 28 in the high-dose arm, of whom 47 were sexually active. Paired samples of symptoms reported during and after tamoxifen were analysed, which showed an improvement in subjective symptoms for all categories 3–6 months after tamoxifen was stopped (Table 4

**Table 4:**
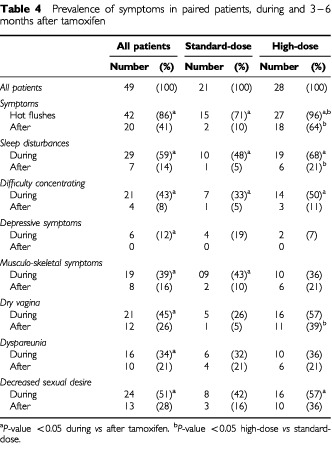
Prevalence of symptoms in paired patients, during and 3–6 months after tamoxifen

). After tamoxifen, hot flushes, disturbed sleep and vaginal dryness were more prevalent in the high-dose group (*P*<0.05). All patients who had reported depressive symptoms during tamoxifen improved after tamoxifen was stopped.

## DISCUSSION

In this study we evaluated subjective side effects and psychosexual function, in relation to menopausal status, in breast cancer patients receiving tamoxifen after standard-dose or high-dose chemotherapy. Although this was not a placebo controlled trial, we are able to draw some conclusions on the impact of tamoxifen use by comparing data from interviews during and after tamoxifen use. As we only included disease-free patients, they may show an improvement in their well-being at the last interview, simply due to the passage of time. To make this time effect as small as possible, we held the last interview shortly (3–6 months) after the discontinuation of tamoxifen.

Adjuvant therapy with daily 40 mg tamoxifen after chemotherapy caused symptoms of which hot flushes and impaired sexual function were most prevalent. Hot flushes was the most frequently reported symptom, not only by postmenopausal but also by premenopausal patients. For comparison, postmenopausal breast cancer patients without adjuvant therapy report hot flushes in about 40% of the cases (Ragaz, 1997). In a recent study of 114 postmenopausal breast cancer patients, hot flushes were reported by 65% of the patients, of which 59% rated the symptoms as severe (Carpenter *et al*, 1998). In our study hot flushes coincided with disturbed sleep and problems concentrating, which was not related to type of chemotherapy or menopausal status. van Dam *et al* (1998) evaluated cognitive function by psycho-neurological tests in patients who were randomly assigned to receive high-dose (*n*=34) or standard-dose chemotherapy (*n*=36) plus 2 years tamoxifen 40 mg daily, and compared the results with those from 34 patients with stage I breast cancer treated with surgery but without systemic therapy. Patients enrolled in their study were at least 6 months post-chemotherapy. The risk of cognitive impairment with high-dose treated patients was 8.2 times higher than in breast cancer patients not receiving systemic therapy and 3.5 times higher than in standard-dose treated patients. The authors concluded that impaired cognitive functioning as defined by a battery of psycho-neurological tests, may be a dose-limiting factor in high-dose chemotherapy. In our study 41% of the patients reported concentration problems during tamoxifen use, however equally distributed over standard-dose and high-dose treated patients. Notwithstanding, concentration problems were related to hot flushes and disturbed sleep and in both treatment groups these symptoms showed an improvement after stopping tamoxifen, suggesting that concentration problems in these patients may, at least in part, be due to tamoxifen. The higher cognitive impairment in the high-dose group in the study of van Dam might be explained by the chemotherapy induced menopause in these patients.

Depressive symptoms were mentioned by 12% of the patients, more often after standard-dose (19%) than high-dose chemotherapy (4%). We hypothesise that this difference between the two treatment groups might be related to menopausal status of the patients and hence the agonistic/antagonistic effect of tamoxifen. As depression and mood-fluctuations may be related to oestrogen-deprivation, depressive symptoms in our study may be related to the anti-oestrogenic effect of tamoxifen in the predominantly premenopausal patients of the standard-dose group, while in the menopausal patients in the high-dose group, tamoxifen acts as an oestrogen agonist. In the placebo-controlled NSABP-prevention-trial, Day *et al* (1999) reported that tamoxifen was not associated with depression in a cohort of healthy women who received 20 mg tamoxifen daily for the prevention of breast cancer. The difference between their data and ours could be a result of the higher tamoxifen dose in our study, as dose reduction from 40 to 20 mg daily in four depressed patients in our study, resulted in amelioration of their depressive symptoms.

Breast surgery and chemotherapy-induced menopause can interfere with self-esteem and sexual function of women (Ganz *et al*, 1992, 1998; Schover, 1994; Meyerowitz *et al*, 1999). This raises the problem of how to measure the effect of tamoxifen on sexual function, considering several causative factors, while no baseline questionnaires are available. Consequently, not the actual level of sexual activity but the changes compared to the situation without tamoxifen treatment are of interest. In order to determine a change in sexual function we evaluated three factors determining sexual function: sexual desire, sexual activity, and pain or discomfort during intercourse. These aspects are also addressed in the recently developed Sexual Activity Questionnaire, which was designed to investigate the impact of long-term tamoxifen use on the sexual function of women in a prevention trial (Thirlaway *et al*, 1996). Forty-four per cent of our patients reported a decreased interest in sexual activity during tamoxifen therapy, unrelated to the type of previous chemotherapy or menopausal status and this might also be an effect of breast cancer diagnosis and the other treatment modalities. However, after tamoxifen was stopped, patients in the high-dose arm reported sexual dysfunction more often (36%) than patients in the standard-dose arm (16%). In early stage breast cancer patients, changes in sexual function are more prevalent in women with ovarian failure due to adjuvant chemotherapy than in women with a premenopausal status (Lindley *et al*, 1998). Ganz *et al* (1998) observed impaired sexual function in women who became postmenopausal, compared to those who retained menstrual cycles, while no effect on sexual function was seen of tamoxifen when added to chemotherapy in the Ganz study, or in another recent study (Berglund *et al*, 2001). On the contrary, Kaplan (1992) suggested that the addition of tamoxifen to chemotherapy exacerbates its sexual side effects. Ganz *et al* (1998) observed impaired sexual function in women who became postmenopausal, compared to those who retained menstrual cycles. In our study, 67% of the patients with induced menopause reported impaired sexual function during tamoxifen, compared to 49% premenopausal patients (data not shown), pointing to an additional effect of induced menopause over the effect of tamoxifen. All except one of these 33 ‘new’ postmenopausal patients had received high-dose chemotherapy. Meyerowitz *et al* (1999) reported that a negative impact from breast cancer diagnosis and treatment on sexuality was most likely in patients who experienced changes in hormonal status. We found a relationship between loss of sexual desire, vaginal dryness and painful intercourse, symptoms that are also frequently reported by healthy women during menopause (McCoy and Davidson, 1985; Greendale *et al*, 1996). In our study however, these symptoms were also reported by premenopausal patients during tamoxifen, suggesting a direct anti-oestrogenic effect of tamoxifen on the vaginal epithelium in these young women.

The relative impact as well as the cumulative impact of adjuvant tamoxifen and chemotherapy on patient well being is an important and neglected topic. Our data indicate that 40 mg tamoxifen daily, following chemotherapy, interferes with psychosexual well-being, causing different side effects of which hot flushes and impaired sexual function are most frequent, both in pre- and postmenopausal women. We suggest that induced menopause due to adjuvant high-dose chemotherapy might explain why symptoms did not fully recover in all patients, after finishing tamoxifen treatment. Therefore, in this study of breast cancer patients younger than 56 years at diagnosis, symptoms are most probably especially due to a combined effect of tamoxifen and the hormonal change induced by high-dose chemotherapy. This is the first study in which side effects are shown to be clustered and it is also the first study about side effects of breast cancer treatment in clearly defined pre- and postmenopausal women, in which menopausal status was defined not only by the last menstrual period but also by serum levels of FSH and oestradiol. Tamoxifen predominantly acts as an anti-oestrogen in premenopausal women, with recovery of symptoms after its cessation. In postmenopausal women tamoxifen predominantly acts as an oestrogen, but is not sufficient to alleviate menopausal symptoms in women with chemotherapy induced menopause.

This knowledge allows the clinician to inform patients about the side effects, including the sexual sequelae, both of tamoxifen use and (high-dose) chemotherapy, and to bring up the subject during follow-up visits. Subjective and psychosexual well-being in breast cancer patients should be further investigated with the ultimate goal of acknowledging the impact of breast cancer therapy, and to develop interventions to minimise side effects.

## APPENDIX 1

### Interview list of self reported symptoms

Vasomotor symptoms

 Hot flushes

 Sweating

 Clammy hands

Fluid retention

 Swollen hands or feet

Sleeping problems

 Difficulty falling asleep

 Difficulty staying asleep

 Nightly awakenings two or

  more times a night

Mood attribute

 Irritability

 Restlessness

 Anxiety/fears

 Concentration problems

Depressed mood

 Feeling miserable and sad

 Lack of enjoyment

 Loss of interest

 Loss of vitality

 Loss of initiative

 Life not worth living

Musculo-skeletal

 Joint pain

 Bone pain

 Muscle cramps

 Muscle stiffness

 Muscle weakness

Gastrointestinal attribute

 Nausea

 Vomiting

 Pyrosis

Dyspepsia

Headaches

Others

## APPENDIX 2

### Interview items about sexual functioning

‘Did you have any sexual activity in the past 6 months?’ Yes/No

If ‘yes’:

*Since tamoxifen*, did you experience:

– Impaired sexual interest Yes/No

– Loss of desire Yes/No

– Vaginal dryness Yes/No

– Painful intercourse Yes/No

– Less frequent sexual activity due to loss of libido Yes/No

– Less frequent intercourse due to pain Yes/No
